# Establishment of a scalable engineered cell-line platform for direct, GMP-grade production of eVLP vectors enabling streamlined generation of gene-edited CAR-T/NK cells

**DOI:** 10.3389/fimmu.2026.1878099

**Published:** 2026-07-27

**Authors:** Wei Lin, Jiaru Shi, Hanyi Chen, Ruikai Chai, Sha Zhu, Lili Chen, Shengshui Mo, Zhengbo Wang, Huaxiu Li, Yaru Feng, Lijun Zhao, Junhui Chen, Guohua Yu, Tao Lu, Jianxun Wang

**Affiliations:** 1 School of Life Sciences, Beijing University of Chinese Medicine, Beijing, China; 2 Shenzhen Cell Valley Biopharmaceuticals Co., LTD, Shenzhen, China; 3 Guangdong Junhou Biopharmaceuticals Co., LTD, Zhongshan, China; 4 Department of Minimally Invasion Intervention, Peking University Shenzhen Hospital, Shenzhen, China; 5 Intervention and Cell Therapy Center, Peking University Shenzhen Hospital, Shenzhen, China

**Keywords:** gene-edited CAR-NK therapy, gene-edited CAR-T therapy, large-scale eVLP preparation, scalable engineered cell-line, self-inactivating retrovirus

## Abstract

**Introduction:**

CRISPR–Cas9 has transformed the engineering of chimeric antigen receptor T (CAR-T) cells and chimeric antigen receptor NK (CAR-NK) cells; however, its clinical translation remains constrained by the high cost, batch-to-batch variability, and stringent regulatory requirements associated with current viral and electroporation-based manufacturing approaches.

**Methods:**

We report an industrial-grade platform based on monoclonal producer cell lines that enables the continuous and scalable generation of engineered virus-like particles (eVLPs) co-packaging Cas9–gRNA ribonucleoproteins (RNPs). A progenitor cell line was established by stably integrating three core modules—Gag-Pol, Gag-Cas9, and the baboon endogenous virus (BaEV) envelope—into a single HEK293T clone. Introduction of a self-inactivating (SIN) retroviral vector encoding the gRNA cassette (exemplified here by CD7) converted this progenitor into a dedicated eVLP producer within 10 days.

**Results:**

Using this platform, we generated CD7-knockout CAR-T/NK cells that retained robust in vitro cytotoxicity, confirming preserved functional activity. Owing to its modular architecture, the platform is readily extensible. For example, integration with Recombinant Adeno-associated Virus (rAAV) donor templates enables site-specific CAR insertion, while multiplexed eVLP cocktails allow simultaneous disruption of multiple genomic loci.

**Discussion:**

It is worth noting that this workflow eliminates the need for electroporation, reduces serum dependency, and significantly lowers the cost of reagent consumables. Collectively, this system provides a GMP-compliant and broadly adaptable strategy for the streamlined manufacturing of next-generation autologous and allogeneic gene-edited CAR-T/NK therapies.

## Introduction

Recent advancements in cancer treatment, specifically CAR-T therapy, have demonstrated significant progress in the management of B-cell lymphoma. The therapeutic efficacy is primarily attributed to the highly expression of two specific antigens, CD19 and BCMA (1, 2), on the surface of the tumor cells. A substantial body of clinical research, targeting to another cancer-specific antigens, has been dedicated to the treatment of acute myeloid leukemia (AML) and solid tumors (3–7). However, the single technology limits the further expansion of CAR-T therapy. For instance, antigens that are highly expressed on T lymphocyte have been utilized as targets for CAR-T cells, which targeting has been demonstrated to induce fratricide (8); The co-incubation with cancer cells frequently results in the induction of PD1 and other protein overexpression, which can promotes immune cell exhaustion, thereby reducing treatment and prognostic outcomes (9–11). Allogeneic universal CAR-T cells (12–14) have emerged as a promising therapeutic modality, offering a cost-effective alternative for patients with limited financial resources and addressing critical unmet clinical needs. Their preparation requires the elimination of graft-versus-host disease (GVHD) and host-versus-graft disease (HVGD) reactions, which can be achieved through targeted knockout of relevant genes, such as HLA-I and TRAC (15). One strategy involves the application of CRISPR technology to disrupt immune cell antigens. Gene editing is commonly implemented via electroporation of Cas9–gRNA ribonucleoprotein (RNP) complexes, which is costly, complicated to operate and causes great damage to cells. Recently, mRNA-loaded lipid nanoparticles (mLNPs) have been developed to efficiently deliver Cas9 mRNA for genome editing (16); however, challenges including suboptimal efficiency in immune cells and off-target activity remain unresolved. To address these limitations, researchers have engineered retrovirus-derived virus-like particles (VLPs) capable of delivering gene-editing proteins and gRNA complexes without carrying nucleic acids that could integrate randomly into the genome (17). This design enhances the safety of gene editing while minimizing off-target effects, representing a highly promising delivery platform. Nonetheless, the large-scale industrial production of engineered VLPs (eVLPs) has not yet been realized, restricting their clinical translation. In this study, we report the stable expression of three essential components—gag-pol, envelope, and gag–gene editor—within a single producer cell line to generate eVLP vectors. Using a self-inactivating retroviral system (18, 19), a targeted gRNA was stably integrated to establish a high-yield, production-stable eVLP cell line. The development of a scalable eVLP manufacturing process offers a robust foundation for the clinical deployment of next-generation cell therapies.

## Materials and methods

### Cell lines and culture conditions

Human embryonic kidney 293T (HEK293T), BaEV-WT64 (from Shenzhen Cell Valley) and Ampho-293 cells were maintained in high-glucose DMEM (Gibco) supplemented with 10% fetal bovine serum (FBS; ExCell) and 1% penicillin–streptomycin (P/S; Gibco). Sub-cultivation was performed at 80% confluence at a split ratio of 1:3–1:5. Jurkat cells were cultured in RPMI-1640 (Gibco) containing 10% FBS and 1% P/S. When the density exceeded 2 × 10^6 cells mL^-1^, half-medium replacement was performed; complete medium exchange was carried out weekly, and cells were re-seeded at 5 × 10^5–1 × 10^6 cells mL^-1^. NK-92 cells were expanded in Procell NK-92 complete medium (CM-0530) and passaged identically to Jurkat cells. Primary human T cells were isolated from peripheral blood mononuclear cells (PBMCs). After thawing, cells were resuspended at 2 × 10^6 cells mL^-1^ in X-VIVO 15 (Lonza) supplemented with 5% FBS, 1% P/S, 100 ng mL^-1^ OKT-3 (Sino Biological) and 500 IU mL^-1^ IL-2 (Sino Biological). After 48 h activation, medium was switched to X-VIVO 15 containing 5% FBS, 1% P/S and 500 IU mL^-1^ IL-2, and cells were maintained as described for Jurkat cells.

### Monoclonal cell line generation

Precursor packaging cell screening: Gag-Cas9-BSD was transfected into BaEV-WT64 cells cultured in a 10cm dish using Lipomaster 2000 (Vazyme). Forty-eight hours post-transfection, medium was replaced with complete medium containing 10 µg mL^-1^ blasticidin S HCl(Gibco). After 1 week, cells were single-cell cloned by limiting dilution into 96-well plates (1 cell per 100 µL) in medium containing 7.5 µg mL^-1^ blasticidin. Wells reaching ~50% confluence were transferred to 6-well plates and maintained under the same antibiotic pressure. Clones at >80% confluence were expanded and cryopreserved; working stocks were subsequently cultured in antibiotic-free DMEM.

SIN-CD7-eVLP producer clone isolation: Clon28 cells stably expressing CD7-gRNA-GFP were subjected to limiting dilution cloning in DMEM complete medium. Monoclonal populations were expanded to 50% confluence in 6-well plates, validated for eVLP production, and cryopreserved.

### Viral-liked particle and retrovirus production

Conventional eVLP: 3.5 × 10^6 HEK293T cells were seeded in 10-cm dishes. After 24 h, transfection was performed using Lipomaster 2000 with 1.1 µg envelope plasmid (VSV-G, GALV or BaEV), 3.375 µg Gag-Pol, 1.125 µg Gag-Cas9 and 4.4 µg gRNA plasmid (targeting AAVS1, TRAC or CD7). Medium was replaced 16 h later, and supernatants were harvested at 48 h, clarified through 0.45 µm filters, precipitated overnight at 4 °C with 10% (w/v) PEG-8000, pelleted at 3000 g for 20 min, resuspended in cold PBS (1/25 of original volume), aliquoted and stored at –80 °C.

BaEV-WT64-derived eVLP: BaEV-WT64 cells were transfected with 4 µg Gag-Cas9 and 6 µg gRNA plasmid; downstream steps were identical to section 4.1.

Monoclonal producer eVLP: 6 × 10^5 precursor monoclonal cells were seeded in 6-well plates. At ≥85% confluence, cells were transfected with 2 µg CD7-gRNA plasmid. After 16 h, medium was replaced, and eVLP-containing supernatant was collected at 48 h, centrifuged (3–000 g, 20 min) and used immediately.

Amphotrophic retrovirus encoding SIN-CD7-gRNA: 3.5 × 10^6 Ampho-293 cells were seeded in 10-cm dishes. At ≥85% confluence, cells were transfected with 10 µg SIN-CD7-gRNA-pSV40-GFP using Lipomaster 2000. Medium was exchanged after 16 h, and virus was harvested at 48 h, filtered (0.45 µm) and stored at –80 °C.

Stable eVLP producer cell line: Cells were expanded to T225 flasks until 100% confluent. Medium was exchanged for DMEM containing 1 mM sodium butyrate, and cultures were shifted to 32 °C. Harvests were performed at 24 h (H1) and 48 h (H2), filtered (0.45 µm), aliquoted and stored at –80 °C.

### Genome-editing validation

T7E1 nuclease assay: PCR primers were designed to flank the target site (± 200 bp on one side, ± 400 bp on the other). Genomic DNA (100 ng) was amplified (30 cycles), and 200 ng of purified product was subjected to T7E1 digestion (NEB) after heteroduplex formation (95 °C 5 min, –2 °C s^-1^ to 85 °C, then –0.1 °C s^-1^ to 25 °C). Reactions were incubated at 37 °C for 15 min, resolved on 2% agarose gels, and band intensities quantified using ImageJ to estimate indel frequencies.

ICE analysis: Amplicons from edited and wild-type samples were Sanger-sequenced. Trace files (.ab1) were uploaded to the Inference of CRISPR Edits (ICE) webtool (https://ice.editco.bio/) to quantify editing efficiencies.

### Flow cytometry

Antibodies used: APC-anti-human TCRα/β (BioLegend 306717), PE-anti-human CD7 (BioLegend 395604), APC-anti-human CD4 (BioLegend 344614), PE-anti-human CD8 (BioLegend 344706), Pacific Blue™-anti-human CD197 (CCR7) (BioLegend 353210), PerCP-anti-human CD45RA (BioLegend 304155), Pacific Blue™-anti-human CD366 (Tim-3) (BioLegend 345042), APC-anti-human TIGIT (VSTM3) (BioLegend 372705), and anti-(G4S)n-B02H1-APC (HyCells GS-ARAP100). Data were acquired on an BD Accuri C6 Plus (BD), CytoFLEX (Beckman Coulter) and analysed with FlowJo v10 and CytExpert 2.6.

### Quantitative PCR for vector copy number

A standard plasmid containing Pol and Cas9 was linearised with EcoRI, yielding 1–310 bp and 2–112 bp fragments; the 1–310 bp fragment encompassing both genes was gel-purified. Ten-fold serial dilutions from 1 × 10^8 to 1 × 10^3 copies plus a no-template control were used to generate standard curves. Genomic DNA samples (40 ng per reaction) were analysed in duplicate using SYBR Green master mix (Vazyme) with primers:

Pol-GC-new-F: 5′-TGAGTATCGGCTACATGAGACCT-3′.

Pol-GC-new-R: 5′-CATGCCCCCGGTTTCCG-3′.

Cas9-F: 5′-CAGATTCGCCTGGATGACCA-3′.

Cas9-R: 5′-ATCCGCTCGATGAAGCTCTG-3′.

Absolute copy numbers were calculated from standard curves and expressed as Cas9/Pol and Pol/RPPH ratios.

### eVLP-mediated gene editing

Target cells were passaged or medium-exchanged at least three times post-thaw; primary T cells were pre-activated for 48 h. Retronectin-coated (Takara) 12- or 6-well plates were pre-loaded with 1 mL eVLP dilution (25 µL concentrated eVLP + 975 µL medium for routine editing; 0.5–25 µL eVLP for dose-titration assays) and centrifuged at 2–500 rpm, 32 °C for 1 h. Target cells were resuspended in the same eVLP suspension, transferred to plates, centrifuged again, and incubated at 37 °C for 2 h before medium replacement. Editing efficiency was assessed at 48–72 h.

### Preparation of CD7 KO+CD7 CAR-T cells

After resuscitation or obtaining fresh PBMC cells, use 100 ng mL ^-1^ OKT-3 (Sino Biological) was activated with 500 IU mL ^-1^ IL-2 (Sino Biological) and cultured for two days to obtain T cells. On the third day, eVLP knockout was performed, and on the fifth day, CAR transduction was performed.

### Cytotoxicity assay and cytokine quantification

CD7 CAR-T cells were normalized to identical CAR positivity by flow cytometry. CFSE-labelled Jurkat target cells (1 × 10^5) were co-cultured with CAR-T at E:T ratios of 4:1, 2:1, 1:1, 1:2,1:4 and 1:8 in 24-well plates (triplicates). After 16 h at 37 °C, cells were stained with Annexin V-APC and PI (Elabscience) and analysed by flow cytometry to determine specific lysis. Supernatants from 1:1 co-cultures were diluted 1:6 and analysed for Granzyme B, TNF-α and IFN-γ using CBA Flex Sets (BD) on a FACS cytometer.

## Results

### The efficiency of eVLP in delivering gene editors to different cells is determined by its own envelope protein, which is consistent with RV

**Figure 1:**
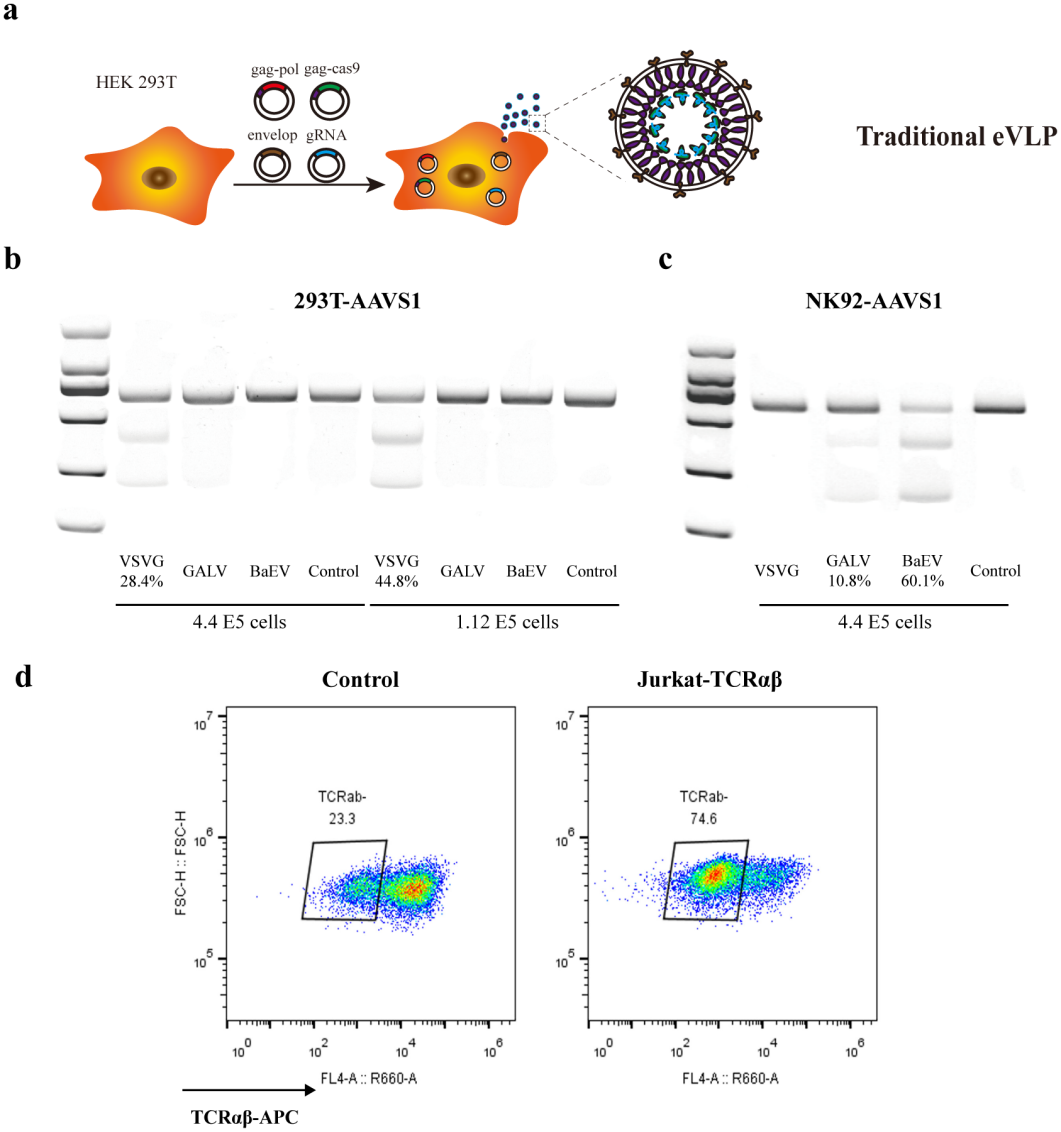
Comparison of targeting efficiency among eVLP vectors with different envelopes and evaluation of knockout efficiency using multiple analytical approaches. **(a)** Schematic representation of the traditional eVLP packaging system. **(b)** T7E1 cleavage assay of eVLP vectors pseudotyped with VSVG, BaEV, and GALV envelopes produced under identical conditions, targeting the AAVS1 locus in 293T cells. **(c)** T7E1 cleavage assay assessing the targeting efficiency of GALV - eVLP and BaEV - eVLP at the AAVS1 locus in NK92 cells. **(d)** Flow cytometry evaluating the knockout efficiency of BaEV - pseudotyped eVLPs at the TCRαβ locus in Jurkat cells.

Given the principal focus of the subsequent application on cell immunotherapy, it is imperative to ensure that the eVLP vectors exhibit a high delivery advantage for T lymphocytes and natural killer cells. As demonstrated in prior studies, the transduction efficiency of retroviruses in various cell types is contingent upon the envelope (20–22). The packaging form of traditional eVLP is shown in **Figure 1a**. Therefore, the initial objective of this study was to ascertain whether the delivery efficiency of eVLP is consistent with that observed in previous retroviral research. We generated eVLPs incorporating three distinct envelope proteins to target the Adeno-Associated Virus Integration Site (AAVS1) locus, a well-recognized genomic “safe harbor” commonly utilized in gene editing owing to its capacity to accommodate transgene insertion without eliciting discernible phenotypic effects (23). In this study, the methods previously identified in various research were employed to prepare eVLPs with different envelopes Vesicular Stomatitis Virus Glycoprotein (VSVG), BAEV, and Gibbon ape leukemia virus Envelope glycoprotein (GALV). Subsequently, the retrovirus transduction method was employed to deliver the Cas9 protein and the gRNA (guide RNA) complex into various cell types. Thereafter, the T7E1 enzyme cleavage assay was utilized to assess the efficacy of the gene editing process (24, 25). As illustrated in **Figures 1b, c**, the VSVG-eVLP exhibited a range of editing efficiencies in 293T cells, with values ranging from 28.4% to 44.8%. However, the editing efficiency at the NK92 AAVS1 site has been determined to be null. In contrast, the BAEV-eVLP demonstrates a remarkable capacity for genome editing in the NK92 cell line, exhibiting an efficiency of 60.1%. Conversely, the GALV-eVLP demonstrates a comparatively lower efficiency of approximately 10.8% in gene editing NK92 cells. In addition, We performed PCR around the gene editing site to produce fragments of approximately 300 to 600 bp, which were then subjected to Sanger sequencing and analyzed for gene knockout efficiency using ICE software (26). Subsequently, a TRAC eVLP was constructed to validate the knockout efficiency in Jurkat cells. The results of the flow cytometry analysis indicate that the efficiency of TCRαβ knockout reached 50% (**Figure 1d**). Moreover, the results of the ICE analysis demonstrated a targeting efficiency of 60%, accompanied by a knockout efficiency of 44% (**Supplementary Figures 1b, c**), which is consistent with the findings of the flow cytometry analysis. This finding suggests that BAEV envelope eVLP can effectively perform genome editing on immune cells, such as natural killer (NK) and T cells. The delivery mechanisms of these eVLPs are analogous to those of Retroviruses(RVs), wherein the envelope protein determines the delivery preference for different target cells. BAEV envelopes have been demonstrated to exhibit enhanced efficiency in the delivery of immune cells, including T, NK, and hematopoietic stem cells (20, 21, 27). Consequently, the BAEV envelope was selected for the construction of subsequent cell lines.

### Preliminary screening of clone 28, which contains three elements: Gag-pol, Gag-Cas9, and BaeV envelope, showed that it can efficiently produce eVLP vectors

**Figure 2:**
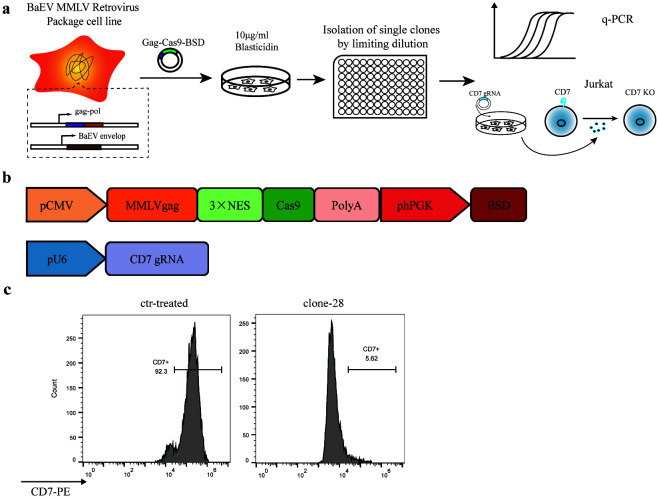
Generation and selection of precursor monoclonal cell lines for eVLP production. **(a)** Schematic representation of the precursor cells used for producing eVLP vectors. **(b)** Schematic representation of plasmid constructs used for establishing precursor monoclonal cells (top) and gRNA plasmid targeting the CD7 locus for functional validation (bottom). **(c)** CD7 gRNA plasmids were transfected into monoclonal cell lines selected in **(b)** to produce eVLPs, and knockout efficiency was subsequently validated in Jurkat cells using the generated eVLPs.

VLP vectors generally comprise four functional modules: Gag-Pol, Gag-Cas9 (gene editor), envelope proteins (optional: VSVG, GALV, amphotropic, ecotropic, BaEV, etc.), and a gRNA for the genome targeting site. To establish a stable and sustainable vector-producing cell factory, all of these components (except the targeting gRNA) must be integrated into a single cell line. After integration, stable expression of the gRNA component can be used to produce specifically targeted eVLP. Previously, our team utilized CRISPR/Cas9 technology to knock out ASCT1/ASCT2 and construct a retrovirus-producing cell line, WT64, which stably expresses the MMLV-Gag-Pol and BaEV envelope (28). Therefore, the stablely introduction of the Gag-Cas9 component into WT64 would create precursor cells for producing eVLP vectors. The schematic diagram is shown in **Figure 2a**.

Gag-Cas9 was originally derived from the pCMV-MMLVgag-3xNES-Cas9 plasmid (Addgene plasmid #181752). The hPGK promoter and blasticidin resistance gene element were then integrated behind Cas9 using a seamless cloning method (29). The resulting plasmid is pCMV-MMLVgag-3xNES-Cas9-PolyA-hPGK-BSD. **Figure 2b** shows the plasmid structure schematic, along with the CD7 gRNA plasmid schematic used for subsequent monoclonal screening. The Gag-Cas9 plasmid was then introduced into the WT64 monoclonal cell line using Lipomaster 2000 (Vazyme, TL201). Forty-eight hours post-transfection, a medium containing 10 μg/mL blasticidin was supplied for selection. After ten days, monoclonal cell lines were isolated using a limiting dilution technique with a seeding density of one cell per well. The cell lines were then cultured continuously in a selection medium containing 7.5 μg/mL Blasticidin, and 42 monoclonal lines were initially selected. Every genomic DNA was extracted, and a quantitative polymerase chain reaction (qPCR) was performed to detect the copy number of Cas9 and Pol elements in the cellular genome (30). The monoclonal lines were screened based on their cellular proliferation status and the element copy numbers. David Liu’s team developed eVLP gene-editing technology that suggests the ratio of Gag-Cas9 to Gag-Pol gene copy numbers within a specific range can significantly enhance gene-editing efficiency. This finding demonstrates that simply increasing the expression level of gag-cas does not necessarily enhance eVLP yield, suggesting that its expression may be optimized within a certain range (17). Accordingly, monoclonal cells exhibiting an Gag-Cas9 to Gag-Pol ratio (Cas9/Pol = 0.4–1.2, Pol/RPPH > 0.7) were selected for further screening. This process led to the isolation of clones 2, 6, 12, 16, 17, 22, 27, 28, 32, 33, and 39. However, clone 47 was discarded due to its slow proliferation, while clone 14 was instead retained for further analysis(**Supplementary Figure 2c**). Subsequently, the CD7 gRNA plasmid was transfected into the 12 monoclonal cell lines, and following a 48-hour period, the CD7 eVLP supernatant was harvested. The supernatants were then used to infect Jurkat cells individually; 48 h post-infection, the knockout efficiency in Jurkat cells was quantified by flow cytometry to identify the high-yield eVLP-producing clones. As illustrated in **Figure 2c**, the employment of the CD7 eVLP derived from the Clon-28 cell line resulted in the 93.16% percentage of CD7-negative Jurkat cells, thereby, Clon-28 exhibits a high degree of efficiency in producing eVLPs when compared with other monoclonal clones.

### SIN-CD7 gRNA-RV can efficiently establish a cell pool that stably produces CD7-eVLPs based on the Clon28 cell line

**Figure 3:**
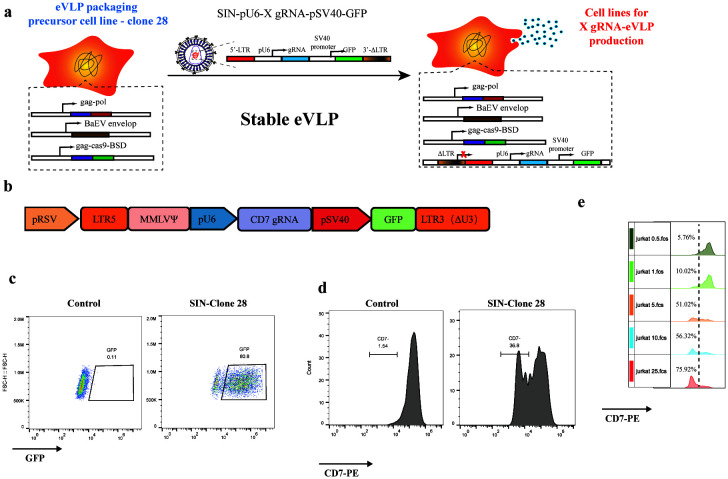
Establishment and validation of stable eVLP-producing cell lines. **(a)** Schematic representation of Stable eVLP generation. **(b)** Schematic diagram of SIN-CD7 gRNA plasmid delivery into precursor monoclonal cells via retroviral vectors. **(c)** Detection of positive transduction rates in precursor monoclonal cell lines following four rounds of SIN-CD7 gRNA retroviral transduction. **(d)** Supernatants collected from different SIN-CD7 eVLP cell lines after equal culture duration in 6-well plates were applied under identical conditions to Jurkat cells for knockout validation. **(e)** The SIN-CD7 eVLP monoclonal cell line (Clone 28) was expanded, and eVLPs were harvested and concentrated 25-fold using PEG 8K, followed by gradient knockout validation in Jurkat cells with increasing doses (0.5μl, 1μl, 5μl, 10μl, 25μl).

A strategy was formulated to achieve stable expression of gRNA elements within a monoclonal cell line. While viral transduction is a widely used approach, when retroviral transduction is employed, integration of a gRNA-containing nucleic acid fragment into the host genome can result in the transcription of a complete 5′LTR–3′LTR structure under the regulatory control of the LTR promoter. Such reconstituted sequences may be repackaged into viral particles, thereby generating retroviruses carrying the gRNA sequence. The eVLP platform was originally developed to avoid introducing nucleic acid fragments with the potential of random genomic integration. In order to mitigate this risk, a self-inactivating (SIN) plasmid vector was employed (18, 19). The 3′LTR was innovatively engineered through partial deletion of the U3 region, ensuring that, upon integration of the viral genome into the host cell, the transferred 3′LTR (ΔU3) loses its promoter activity. This modification effectively prevents the reassembly of a functional LTR structure, thereby inhibiting the repackaging of the delivery plasmid (**Figure 3a**). In accordance with this principle, the SIN-CD7 gRNA plasmid (**Figure 3b**) was engineered, incorporating a green fluorescent protein (GFP) reporter gene driven by the SV40 promoter, located downstream of the gRNA element.

Monoclonal cell lines—Clones 28—were selected for the parallel generation of stable producer lines. Initially, SIN-CD7 gRNA retrovirus was packaged in Ampho 293 cells and subsequently used to transduce the monoclonal clones. Green fluorescent protein (GFP) expression was assessed by flow cytometry 48 hours post-transduction, Clones 28 surpassing 80% (**Figure 3c**). Culture supernatants containing eVLP from each clone were then collected and used to transduce Jurkat cells. As shown in **Figure 3d**, eVLP derived from Clone 28 exhibited knockout efficiency. Based on its superior performance, Clone 28 was selected as the parental producer line for eVLP vectors expressing both the BaEV envelope protein and spCas9 nuclease. The SIN-CD7 eVLP cell line, derived from Clone 28, was subsequently expanded in T225 flasks. The culture supernatant was harvested, concentrated 25-fold, aliquoted, and stored at –80 °C. Dose–response knockout assays were then performed by coating plates with 0.5, 1, 5, 10, or 25 µL of concentrated eVLPs and transducing Jurkat cells with the corresponding volumes. The resulting knockout efficiency (**Figure 3e**) demonstrated that 25 µL of eVLP from the SIN-CD7 eVLP line achieved a 76% knockout rate in Jurkat cells, while even 0.5 µL yielded a 6% knockout rate. Collectively, these results indicate that the stably producing eVLP vector possesses robust and efficient genome-editing activity.

### The SIN-gRNA-GFP nucleic acid was not repackaged in the stably produced eVLPs and after knockout in T cells, the performance was consistent with the eVLPs prepared in 293T

**Figure 4:**
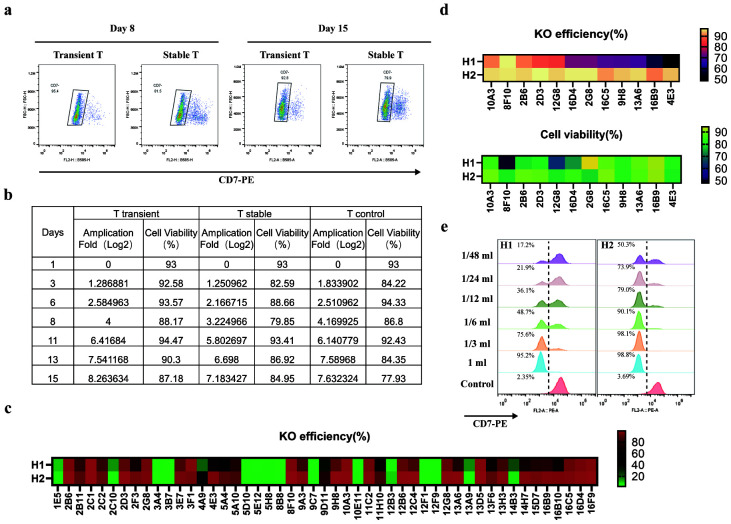
Clone 10A3, screened from cells stably producing eVLPs, demonstrates the advantages of more stable, higher titer, and no cell damage in eVLP transducton. **(a, b)**. Stably produced eVLPs exhibit comparable performance to transient eVLPs in T cell CD7 knockout, including CD7 knockout efficiency determined by flow cytometry **(a)**, cell proliferation assessed by cell counts, and cell viability measured by trypan blue staining **(b)**. **(c)** Single-cell clones were isolated by limiting dilution, yielding 49 monoclonal lines. Two batches of eVLP supernatants were collected and tested for CD7 knockout efficiency in Jurkat cells. **(d)** Twelve monoclonal lines selected from the initial screen were further evaluated by collecting two batches of eVLP supernatants and performing CD7 knockout in primary T cells. CD7 knockout efficiency was assessed by flow cytometry at 72 h(above), and T-cell viability was determined by trypan blue staining(below). **(e)** Clone 10A3 was identified as the optimal candidate, expanded for large-scale culture, and used to produce two batches of GMP-grade CD7-eVLPs, H1 and H2. Gradient transductions (1/48, 1/24, 1/12, 1/6, 1/3, 1 ml) were performed in Jurkat cells to generate a knockout efficiency histogram.

Subsequently, the objective was to determine whether LTR-associated nucleic acid structures were incorporated into the eVLP vector. The fundamental safety validation principle of this system is based on the following rationale: if an LTR–U6–gRNA–SV40–GFP–LTR nucleic acid sequence were inadvertently packaged into the eVLP, transduction of target cells would lead to green fluorescent protein (GFP) expression driven by the SV40 promoter. As shown in **Supplementary Figure 3**, Jurkat cells, transduced by 25 μL of stably produced eVLP, achieved an 88% CD7 knockout efficiency, as measured by CD7–PE IgG staining. In contrast, the GFP-positive rate in target cells was only 0.23%, well below the threshold for a positive signal and consistent with the results without antibody staining. These findings support three key conclusions: 1) The incidence of LTR-associated nucleic acid contamination in the produced eVLP vector is effectively negligible. 2) The self-inactivating design reliably prevents potential repackaging of the LTR-gRNA element. 3) The vector system meets the safety criterion of “no risk of genomic integration”. This safety validation strategy establishes a stringent quality control benchmark for the clinical translation of genome-editing therapies. Following safety verification, a systematic functional comparison was performed between the stable production system (Stable eVLP) and the conventional four-plasmid transient transfection system (Transient eVLP), evaluating genome-editing efficiency and stability. Primary T cells served as the target model, and CD7 knockout efficiency was quantified by flow cytometry. As shown in **Figure 4a**: 1) Editing efficiency analysis revealed that the stable production system achieved an 80% knockout efficiency, slightly lower than the 92.9% observed with the transient system. 2) Longitudinal stability assessment on days 8 and 15 post-transduction showed minimal efficiency loss in both systems, with the transient system decreasing from 95.4% to 92.9% and the stable system from 81.5% to 80%. T-cell proliferation and viability after CD7 knockout were assessed in **Figure 4b**, comparing transient and stable eVLP production. In the stable knockout group, cell viability remained above 80% and proliferation was slightly reduced before day 8 (approximately 10% below the peak values of other groups). However, both parameters recovered thereafter, with no obvious differences from the other groups.

Collectively, these results demonstrate that, while the stable production system exhibits a modestly lower peak editing efficiency, it delivers high, consistent knockout performance without detectable cytotoxicity. Owing to its superior process stability and reproducibility, the stable production system is particularly well suited for clinical-grade manufacturing of genome-editing vectors.

### Clon 10A3 isolated from the SIN-CD7-eVLP cell pool was used for industrial stable and scalable production

To facilitate efficient, safe, and stable large-scale industrial production of CD7 eVLP, a systematic monoclonal isolation and functional evaluation of the SIN-CD7 eVLP cell line was undertaken. Monoclonal sorting was performed using the limiting dilution method, yielding 49 candidate clones in the initial screening round. Following expansion, two independent batches of eVLP-containing supernatant were harvested from each clone under identical culture conditions to those used during the initial phase. These batches were subsequently used to transduce Jurkat cells, as shown in **Figure 4c**. Preliminary flow cytometric analysis of CD7 knockout efficiency identified 12 promising monoclonal candidates. eVLPs derived from these 12 clones were next evaluated in primary T cells. Among them, clone 10A3 consistently generated two independent eVLP batches (designated H1 and H2) that each achieved >90% CD7 knockout efficiency in T cells. Importantly, post-editing T-cell viability remained consistently above 85% (**Figure 4d**). Comparative analysis demonstrated that 10A3 outperformed all other candidates in both editing efficiency and cell viability. Subsequently, the 10A3 monoclonal strain was used to produce GMP-grade eVLP vectors, and gradient knockout verification was performed (stock solution, 1/3, 1/6, 1/12, 1/24, 1/48), which proved that the dose of eVLP was positively correlated with the knockout efficiency. It was also found that the eVLP produced by H2 still had a knockout efficiency of more than 90% for 2×10 (5) Jurkat cells after being diluted 3 times (**Figure 4e**). Consequently, clone 10A3 was selected as the optimal monoclonal producer cell line for large-scale, industrial-grade manufacturing of CD7 eVLP vectors.

### Establishment of a strategy for the preparation of CD7 knockout and CD7 CAR -T through optimization of timing and envelope RV

The eVLP vector was employed to address fratricide in CD7 CAR-T cells, adapting a previously established strategy in which CD7 is first disrupted, followed by transduction with a CD7 CAR construct (8). Unlike conventional approaches that typically use electroporation for CD7 knockout prior to CAR transduction (31, 32), this study implemented eVLP-mediated knockout as a non-electroporation alternative. Guided by this framework, process optimization was conducted along two dimensions: timeline and vector envelope. Timeline optimization included three experimental groups and one control group [(**Supplementary Figure 4a**): 1] CAR transduction at 24 hours post-eVLP-mediated CD7 knockout. 2) CAR transduction at 48 hours post-eVLP-mediated CD7 knockout. 3) Simultaneous CD7 knockout and CAR transduction. 4) CD7 CAR group without knockout served as the control. Vector optimization involved two retroviral backbones encoding the CD7 CAR and pseudotyped with either GALV or BaEV envelopes (**Supplementary Figure 4d**).

**Figure 5:**
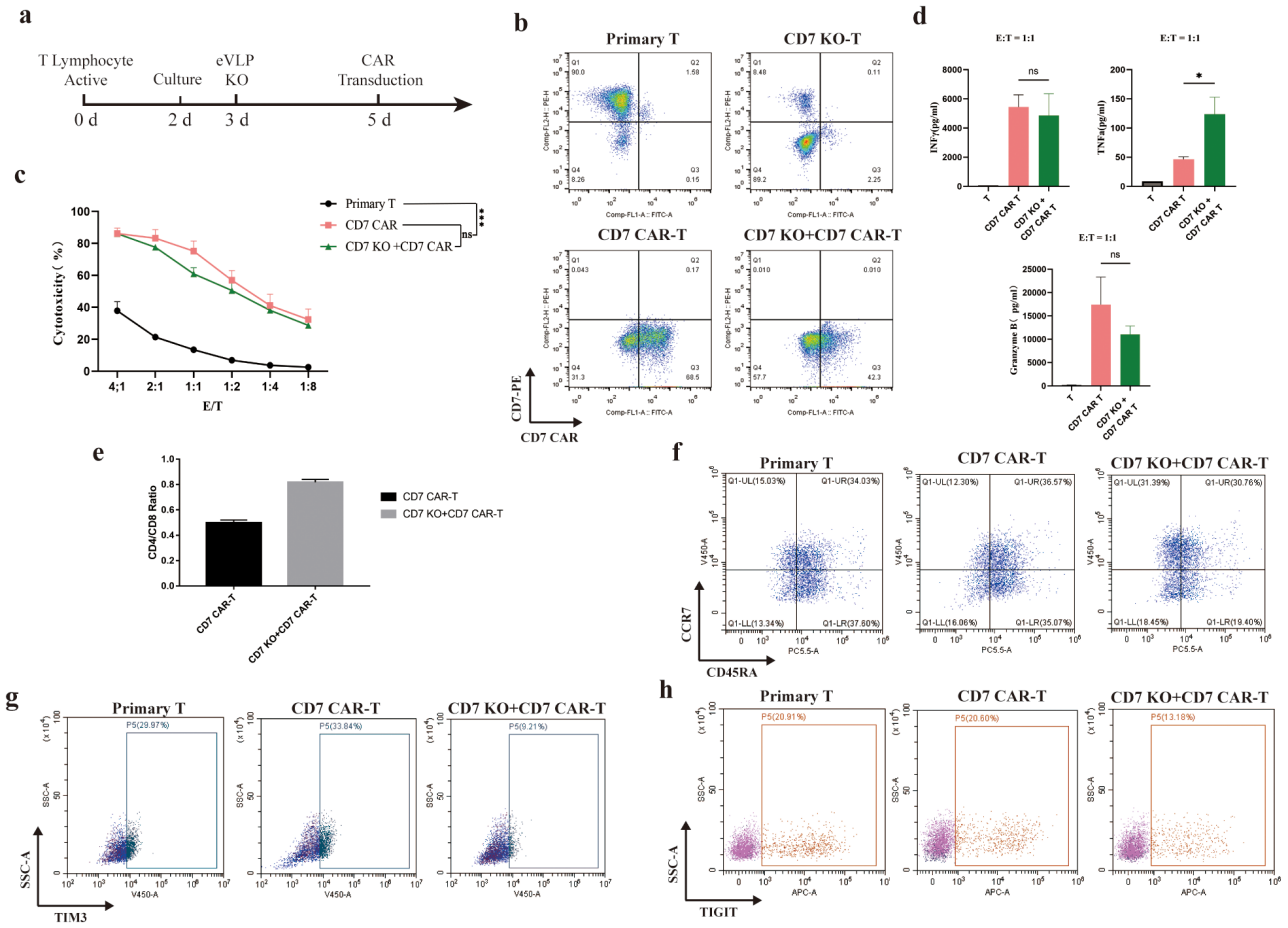
Generation and Functional Validation of 1×10^8 CD7-KO/CD7-CAR-T Cells Derived from Healthy-Donor PBMCs. **(a)** Schematic diagram of CD7 KO + CD7 CAR-T cell preparation. **(b)** Flow cytometry analysis of knockout efficiency and CAR expression in CD7 KO + CD7 CAR-T cells. **(c)** Comparison of cytotoxicity curves against Jurkat cells at different effector-to-target (E:T) ratios between naturally selected CD7 CAR-T cells and CD7 KO + CD7 CAR-T cells. (n=3) **(d)** At an E:T ratio of 1:1, secretion levels of Granzyme B, TNFα, and IFNγ were compared between the two CAR-T cell groups after 16 h co-culture with Jurkat cells. (n=3) **(e)** The CD4 and CD8 ratios of CD7 CAR-T cells and CD7 KO + CD7 CAR-T cells. (n=3) **(f)** Memory phenotype detection of CD7 CAR-T cells and CD7 KO + CD7 CAR-T cells. **(g)** Depletion phenotype TIM3 detection of CD7 CAR-T cells and CD7 KO + CD7 CAR-T cells. **(h)** Detection of TIGIT expression in exhausted phenotypes of CD7 CAR-T cells and CD7 KO + CD7 CAR-T cells. c, **(d)** Values were expressed as the means ± SD, unpaired t test was performed. ***p<0.001; **p<0.01; *p<0.05.

As shown in **Supplementary Figure 4b**, all three knockout groups achieved >90% CD7 disruption by day 9. The CAR positivity rate was 52.2% in the 24-hour group and 35.7% in the 48-hour group—both exceeding the clinical threshold of 30%. In contrast, the simultaneous knockout–transduction group achieved only 15.5% CAR-positive cells, indicating suboptimal manufacturing efficiency. Proliferation kinetics (**Supplementary Figure 4c**) revealed that the 48-hour knockout-to-CAR transduction schedule was therefore selected as optimal. The single-transduction CD7 CAR group expanded most slowly, previous research suggested that CD7 CAR expression can block endogenous CD7, enabling CAR^+^ cells to evade self-killing while effectively killing CD7^+^ cells that were not transduced with CAR (32). However, in groups with low CAR positivity, this killing effect was insufficient to counteract the natural expansion of CD7^+^ cells, leading to ongoing fratricide, impaired CAR^+^ expansion, and minimal overall proliferation. In subsequent large-scale manufacturing of high-CAR-positivity CD7 CAR-T cells (**Figure 5b**; **Supplementary Figure 5a**), the single CAR transduction schedule yielded a final product (day 16) composed entirely of CD7^–^ cells with 68.5% CAR positivity, no detectable fratricide, and restored proliferation.

When BaEV-pseudotyped CD7 CAR retrovirus was used in multiple KO + CAR production runs, post-manufacturing cell viability consistently fell below 40%, with negligible proliferation. As a result, the BaEV-CD7-eVLP and BaEV-CD7-CAR combination was discontinued. However, in order to determine whether the defect arose from the retroviral backbone or the BaEV envelope (given the fratricide observed in single-transduction CD7 CAR T cells), BaEV- and GALV-pseudotyped control CAR retroviruses (NEF-CTR CAR) were tested in single-transduction experiments. Both vectors achieved >50% CAR positivity and proliferation comparable to Primary T cells (**Supplementary Figures 4d, f**). This analysis was extended to the CD7-eVLP and CAR-RV workflow using T cells with ~100% CD7 knockout, four CAR retroviruses were tested: BaEV-NEF-CD7, GALV-NEF-CD7, GALV-CD7, and BaEV-NEF-CTR. As shown in **Supplementary Figure 4e**, all achieved >40% CAR positivity. However, **Supplementary Figure 4g** demonstrates that BaEV-pseudotyped CAR retroviruses induced a pronounced impairment in cell proliferation compared with aforementioned BaEV-NEF-CTR–transduced T cells, which had not undergone prior knockout. In contrast, GALV-pseudotyped CAR retroviruses supported robust expansion, with GALV-NEF-CD7 T cells achieving a 55-fold increase during 15 days period.

Collectively, these findings established the final manufacturing workflow: BaEV eVLP-mediated CD7 knockout followed 48 hours later by GALV-pseudotyped CD7 CAR transduction. While the precise mechanism underlying the BaEV CAR defect remains unclear, it is plausible that BaEV-pseudotyped eVLPs deplete ASCT1/2 receptors from the T-cell surface, thereby impairing subsequent BaEV-pseudotyped CAR transduction.

### CD7 KO+CD7 CAR-T cells showed consistent cell proliferation and cytotoxicity compared to CD7 CAR-T cells previously used in clinical studies

Peripheral blood mononuclear cells (PBMCs) from healthy donors were used for large-scale manufacturing of CD7 KO + CD7 CAR T cells. T cells were activated for 48 hours and subsequently seeded at a density of 2 × 10^6^ cells/well to initiate production. The BaEV eVLP + GALV CAR retroviral vector (RV) protocol was implemented, with proportional scaling of viral input. A single-transduction CAR group served as the control. For each group, 5 × 10 (7) CAR-T cells were generated.

On day 16, flow cytometric analysis (**Figure 5b**) showed CD7 knockout efficiencies exceeding 90% in both groups. The CAR-positive fraction was 68.5% in the CD7 CAR-only group and 42.3% in the CD7 KO + CD7 CAR group, both surpassing the clinical usage standards (>30%) for adoptive cell therapy. Proliferation kinetics (**Supplementary Figure 4g**) revealed that the CD7 CAR-only group exhibited reduced early expansion due to fratricide; however, by day 16, the population had fully converted to CD7^-^ cells, proliferation was restored, and cumulative expansion matched that of the CD7 KO + CD7 CAR group, consistent with previous observations (32). Cytotoxicity assays (**Figure 5c**) were performed against Jurkat target cells at varying effector-to-target (E:T) ratios following 16 hours of co-culture. Killing activity was indistinguishable between groups, aligning with prior findings. Cytokine release analysis at a 1:1 E:T ratio (**Figure 5d**) confirmed that both manufacturing protocols yielded functionally competent CAR-T cells. The **Figure 5d** indicates a significant increase in TNFa cytokine expression in the CD7 KO+CD7 CAR-T group, while the trends of INFγ and Granzyme B were consistent with the killing curve and there was no significant change. The diminished TNFα expression observed in the CD7 CAR-T group in this study may be attributable to the blocking of the CD7 target by the CAR molecule. However, the role of TNFa in tumor progression remains controversial, and further investigation in animal models of T-cell lymphoma will be required to confirm the unique efficacy of these prepared CAR-T cells. On day 7, CD7 KO+CD7 CAR-T has a higher CD4/CD8 ratio (**Figure 5e**). The CD7 KO+CD7 CAR-T has a higher population of central memory T cells and effector memory T cells (**Figure 5f**), suggesting that it can generate better immune memory. Compared to the experimental group without knocking out CD7, the depletion phenotype Tim3 and TIGIT of CD7 KO+CD7 CAR-T were reduced (**Figures 5g, h**), confirming that CD7 is the driving force behind T cell depletion phenotype. This may be achieved by amplifying T cell receptor (TCR) signals and inducing the expression of depletion related transcription factors through CD7, and can be slowed down by knocking out CD7 to alleviate T cell depletion.

## Conclusion

This study established a precursor cell line capable of producing BaEV-envelope–pseudotyped vectors for Cas9 delivery. Subsequently, self-inactivating viral vectors were employed for gRNA delivery, streamlining the generation of stable producer cell lines, markedly reducing preparation time, and effectively eliminating potential interference from multiple antibiotic resistance elements. Based on this approach, we developed a robust strategy for the stable production of target-specific eVLP. Implementation of this strategy for CD7-targeted eVLP confirmed their high efficiency and safety. Leveraging this system enables large-scale construction of producer cell lines for diverse eVLP targets, thereby establishing a solid foundation for clinical translation.

The eVLP platform developed herein integrates the BaEV envelope to confer efficient transduction of hematopoietic cell lines and offers broad applicability. It can be deployed individually for site-specific gene knockout or in combination with multiple eVLPs to achieve multiplexed gene disruption, demonstrating exceptional flexibility. When paired with AAV-mediated donor DNA delivery, it supports precise gene knock-in, providing a practical framework for safer and more efficient CAR-T/NK cell manufacturing.

Furthermore, the cell line generation strategy is readily adaptable to various gene editing modalities, including base editors, dCas9, nCas9, and Cas12a (17, 33, 34). It is also extensible to alternative envelope-pseudotyped eVLP for efficient delivery into non-hematopoietic cell types. Notably, the platform is compatible with state-of-the-art prime editing (35–37), facilitating the creation of prime editing–specific eVLP producer lines. Collectively, this system addresses a critical bottleneck in the clinical translation of gene editing technologies, effectively bridging the “last mile” from bench to bedside.

## Data Availability

The original contributions presented in the study are included in the article/**Supplementary Material**, further inquiries can be directed to the corresponding author/s.
