# Supplementary material for: Establishment of a scalable engineered cell-line platform for direct, GMP-grade production of eVLP vectors enabling streamlined generation of gene-edited CAR-T/NK cells
Source: Front Immunol. 2026 Jul 27;17:1878099. doi: 10.3389/fimmu.2026.1878099 (PMC13454236; doi:10.3389/fimmu.2026.1878099)
Supplement: Supplementary file 1 [file Table1.docx]

**Supplement Fig 1. Flow cytometry detection of Jurkat cell knockout efficiency of eVLPs with different envelopes. a. Flow cytometry evaluation of the effect of different envelope proteins on CD7 knockout efficiency in Jurkat cells. b. ICE analysis assessing the targeting efficiency of GALV - eVLP and BaEV - eVLP at the AAVS1 locus in NK92 cells. c. ICE analysis evaluating the knockout efficiency of BaEV - pseudotyped eVLPs at the TCRαβ locus in Jurkat cells.**

**Supplement Fig 2. Evaluation of precursor monoclonal cell line generation and screening using qPCR. a. Knockout and qPCR Primer Table. b. Detection of dissolution curves of different gene fragments with copy numbers using qPCR. c. Copy numbers of Cas9 and Pol elements in monoclonal cells derived from gag-Cas9 plasmid transfection were quantified by qPCR, with RPPH as the internal reference; Cas9/Pol ratios and relative Pol copy numbers were calculated.**

**Supplement Fig 3. Verification of the self-inactivation property of the virus by transduction of Jurkat cells with prepared eVLPs. After transducing Jurkat cells with 25 μl of stably produced eVLPs, the knockout efficiency was determined by staining with a CD7-PE antibody, and GFP expression was used to determine whether the nucleic acid sequence from the self-inactivating virus had been introduced into the eVLPs.**

**Supplement Figure 4: Establishment of a strategy for CD7 knockout and CD7 CAR delivery through optimization of timing and envelope vectors. a. T cells were subjected to eVLP-mediated CD7 knockout at 0, 24, or 48 h prior to CD7 CAR transduction, and knockout efficiency was assessed 72 h later. b. Cell proliferation curves were plotted for groups with different knockout–transduction timing schedules. c–d. T cells were transduced with CTR-CAR retroviruses pseudotyped with GALV or BaEV envelopes (to avoid fratricide). CAR-positive rates were measured by flow cytometry at 72 h (c), and proliferation curves were recorded (d). e–g. T cells pretreated with eVLP-mediated CD7 knockout for 48 h were transduced with BaEV-CD7-CAR, GALV-CD7-CAR, or BaEV-CTR-CAR; non-knockout T cells transduced with GALV-CD7-CAR served as controls. Knockout and transduction efficiencies were analyzed (e), and proliferation curves were compared across groups (f, g).**

**Supplement Figure 5:** **Flow cytometry was used to evaluate the knockdown efficiency of multiple targets in eVLP, TRAC eVLP was prepared using only TRAC gRNA to knock out T cells; CD7 eVLP is an eVLP knockout T cell prepared using only CD7 gRNA; CD7&TRAC eVLP is a dual target eVLP knockout T cell prepared in a 1:1 ratio of TRAC gRNA and CD7 gRNA; CD7 eVLP+TRAC eVLP is a mixture of eVLP prepared using only TRAC gRNA and eVLP prepared using only CD7 gRNA in a 1:1 ratio to knock out T cells**
